# Local Chemical Gradients in a Mammalian Cortex Measured In Vivo With a Silicon Nanodialysis Mass Spectrometry Platform

**DOI:** 10.1002/anie.3609448

**Published:** 2026-05-06

**Authors:** Weihua Shi, Keyin Li, Yu Ding, Alex G. Armstrong, Jonathan V. Sweedler, Yurii Vlasov

**Affiliations:** ^1^ Department of Electrical and Computer Engineering University of Illinois Urbana Champaign Urbana Illinois USA; ^2^ Neuroscience Program University of Illinois Urbana Champaign Urbana Illinois USA; ^3^ Department of Chemistry, University of Illinois Urbana Champaign Urbana Illinois USA; ^4^ Beckman Institute for Advanced Science and Technology, University of Illinois Urbana Champaign Urbana Illinois USA; ^5^ Chan Zuckerberg Biohub Chicago Illinois USA; ^6^ Carle Illinois College of Medicine University of Illinois Urbana Champaign Urbana Illinois USA

**Keywords:** in vivo sampling, mass spectrometry, neurochemistry, neurotransmitters

## Abstract

Chemical extrasynaptic signaling in the mammalian brain is involved in the control of behavior via modulation of neural activity, in wiring the brain by directing the axonal growth, in localization of pharmacological effects of drugs, and in regulating the neuroinflammation. Local gradients of various neurochemicals in the brain are difficult to study in vivo due to their complex spatiotemporal dynamics induced by intricate interactions between neurons and glial cells that are not well understood. Here, to directly measure in vivo gradients of multiple neurotransmitters and metabolites simultaneously, we utilize an open‐flow silicon nanodialysis sampling platform coupled with sensitive mass spectrometry. Results reveal strong millimeter‐scale spatial gradients in concentration of neurotransmitters, neuromodulators, and astroglial modulators in a mouse cortex. Formation and maintenance of such local chemical compartments indicate strong regulation of brain neurochemistry by glial‐neuron interactions that may heavily influence physiological and pathophysiological modulation of brain functions.

## Graphical abstract

Fluorescence image of the coronal slice of mouse brain where multiple neurochemicals have been measured in vivo with a nanodialysis platform coupled to CE‐MS to reveal strong millimeter‐scale spatial chemical gradients. Formation and maintenance of such local chemical compartments in the otherwise diffusion‐homogeneous cortex indicates strong regulation of ECS neurochemistry imposed by glial‐neuron interactions that may heavily influence physiological and pathophysiological modulation of neural computations.

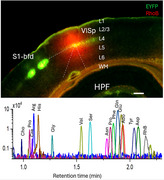

## 1 Introduction

The mammalian brain is heterogeneous, with strong gradients in cytoarchitectonics, gene expressions, structural connectivity, and functional interactions at long‐range scales separating different anatomical areas, as well as locally across cellular dimensions [1]. Local chemical gradients of neurotransmitters, neuromodulators, metabolites, hormones, and paracrine ligands in the brain's extracellular space (ECS) augment this heterogeneity and are involved in control of behavior via modulation of neural activity [2], in wiring the brain by directing axonal growth [3], in localization of pharmacological effects of drugs [4], and in directing immune cell migration in neuroinflammatory response [5]. Local chemical gradients in microenvironment niches additionally underlie growth, invasion, and recurrence of brain tumors [6].

While fundamental to understanding brain function in health and disease, the local chemical gradients in the brain ECS are difficult to measure in vivo due to their complex spatiotemporal dynamics induced by combined effects of synthesis, secretion, uptake, and metabolism of multiple interacting molecules. Recent progress in the development of genetically engineered fluorescent [7] and electrochemical [8] biosensors has demonstrated sub‐second temporal resolution with high chemical sensitivity; however, these approaches are typically limited to only a select few neurochemicals. Multiplexed sampling techniques that enable enhanced chemical detail, such as microdialysis (MD) [9] and push‐pull perfusion (PPP) [10, 11, 12], usually have poorer temporal and spatial resolution due to a fundamental tradeoff between chemical sensitivity and spatial and temporal resolution [13, 14]. In standard commercial MD probes, the sampling surface area *S* is as large as 10^8^ µm^2^, effectively averaging chemical content over large brain volumes, preventing detection of local gradients. The large MD probe cross‐section (10^5^ µm^2^) also results in inevitable tissue trauma during implantation that complicates chemical analysis. Recent advances in miniaturization of MD probes using microfabrication technologies [15, 16, 17, 18, 19] have begun to address these issues by decreasing the flow rates to 100 nL/min and below [16, 19], enabling significant reduction of the sampling area down to just a few thousand µm^2^. Together with integration with droplet microfluidics [17, 18, 19], this results in significantly improved temporal resolution, although it adds complexity to follow‐up chemical assays. We have recently demonstrated a silicon nanodialysis (ND) sampling platform [20, 21, 22, 23, 24] with drastically reduced ultra‐slow nL/min flow rates that enables highly localized chemical sampling with spatial resolution as small as 100 µm while maintaining high analyte recovery rates (*RR*) [21] reaching attomoles in limits of detection [24].

Here, we utilize our ND probe concept to directly measure chemical gradients of multiple neurotransmitters and other metabolites simultaneously across millimeter‐scale distances in an undamaged live mouse cortex. Coupling an open‐flow ND sampling strategy with capillary electrophoresis‐mass spectrometry (CE‐MS) allows for the quantification of neurochemicals in sub‐microliter dialysate samples. Results reveal strong millimeter‐scale spatial gradients in extracellular concentration of neurotransmitters such as glutamate (Glu), neuromodulators such as proline (Pro), and astroglial modulators such as adenosine (Ado). Formation and maintenance of such local chemical compartments [25] in the otherwise diffusion‐homogeneous cortex indicates strong regulation of ECS neurochemistry imposed by glial‐neuron interactions [26, 27, 28] that may heavily influence physiological and pathophysiological modulation of neural computations [29, 30].

## 2 Results and Discussion

### 2.1 Open‐Flow ND Probe Design for Ultra‐Localized Sampling and High Recovery With Ultra‐Slow Flows

**FIGURE 1:**
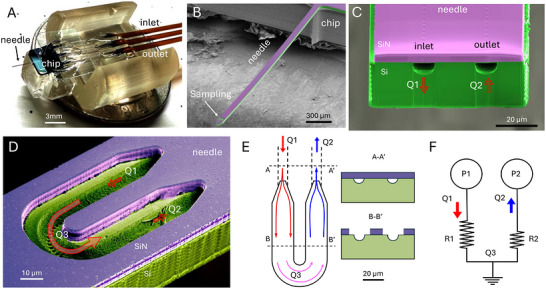
Design and fabrication of open‐flow nanodialysis probe for localized sampling. (A) Photograph of the packaged ND probe. (B) SEM view of the probe needle. (C) SEM view of the needle cross‐section showing buried microfluidic channels. (D) SEM of the open‐flow sampling area. (E) Design of the open‐flow sampling area with two cross‐sections. (F) Equivalent circuit diagram of the pressure‐controlled microfluidic flows.

To measure chemical gradients of multiple neurochemicals in a live brain, we designed a silicon ND probe [21] with a base silicon chip containing inlet/outlet channels for microfluidic packaging and an integrated 3‐mm long needle for implantation into the brain and local sampling (Figure 1A). The ND probe is fabricated via a wafer‐scale microfabrication process [20, 21, 22, 23, 24] (Supplementary Information, SI) that involves 6 aligned photolithography levels and 12 etching/deposition steps to yield a needle (Figure 1B) with a cross‐section of just 70 × 25 µm^2^ (Figure 1C). A pair of 5 µm‐radius parallel microfluidic channels are carved into a silicon layer and are sealed on‐top with a silicon nitride (SiN) cap (Figure 1C).

To enable highly localized sampling while maintaining high recovery rates, we adopted a membrane‐free open‐flow dialysis concept (Figure 1D). In traditional MD, the membrane serves two purposes: to isolate internal perfusate flows from directly entering into the tissue, minimizing flow‐induced tissue damage [12], and cutting‐off large proteins from diffusing into the probe to avoid downstream clogging. The immediate drawback is additional membrane resistance (*R_m_*) for molecular mass transport from the tissue into the perfusate (Equation 1), which degrades the probe recovery rate (*RR*) [13, 14], as:

(1) $$RR=\;\frac{C_{dial}-C_{perf}}{C_{ECS}-C_{perf}}=1-exp\left[-\frac{S}{Q\left(R_{d}+R_{m}+R_{ECS}\right)}\right]$$

where *S* is the active sampling area of the probe, *Q* is the perfusate flow rate, and *R_d_*, *R_ECS_* are the mass transport resistances for the dialysate, and the tissue, respectively. *C_dial_*, *C_perf_*, and *C_ECS_* are the concentrations of analyte in the dialysate, in the perfusate (typically zero), and in the undisturbed ECS tissue, respectively. Even with the ultra‐thin 30 nm silicon membrane [21] used at ultra‐slow *Q* of just 10nL/min, the *RR* is degraded from 80% to 10%. Therefore, if the downstream clogging can be controlled, the membrane can potentially be completely eliminated by redesigning the sampling area to avoid tissue‐directed flows.

Therefore, to eliminate turbulent out‐of‐plane perfusate flow directed into the tissue that is typical for PPP [10, 11, 12], we adopt a conceptually different open‐flow membrane‐less (*R_m_* = 0) dialysis approach (Figure 1D) in which the sampling area is defined by etching out the top SiN cap at the channel U‐turn close to the needle tip (Figure 1E). To maintain pressure in the open sampling area close to 5 mbar intracranial pressure [31], the perfusate (*Q*_1_) and dialysate (*Q*_2_) flows (Figure 1F) inside the inlet and outlet channels (*R*_1_ and *R*_2_ hydrodynamic resistances) are carefully balanced by applying matching pressures (*P*_1_ and *P*_2_, respectively). To contain the flow at the sampling area *Q*_3_ within a probe plane, the inlet and outlet 10 µm‐wide channels are gradually tapered to a 20 µm‐wide U‐turn section. Here the laminar perfusate flow slows down 1.8 times (defined by the ratio of corresponding cross‐sections in Figure 1E) and rinses the exposed brain tissue mostly tangentially to maintain virtually zero net liquid exchange (Figure 1D). The analyte recovery is therefore driven only by diffusion in the sampling area *S* of 4200 µm^2^ that provides highly localized sampling within its 100 µm length. To maintain high *RR* of Equation (1) for this open‐flow ND design, in which the area *S* is reduced 200‐fold in comparison with typical MD, besides eliminating the membrane resistance (*R_m_* = 0), the flow rate *Q*_3_ is reduced to just a few nL/min, which is 100‐fold slower than in typical low‐flow MD or PPP.

### 2.2 Tissue Damage During Implantation and Operation of Open‐Flow ND Probe Is Fewer Than Ten Cells

**FIGURE 2:**
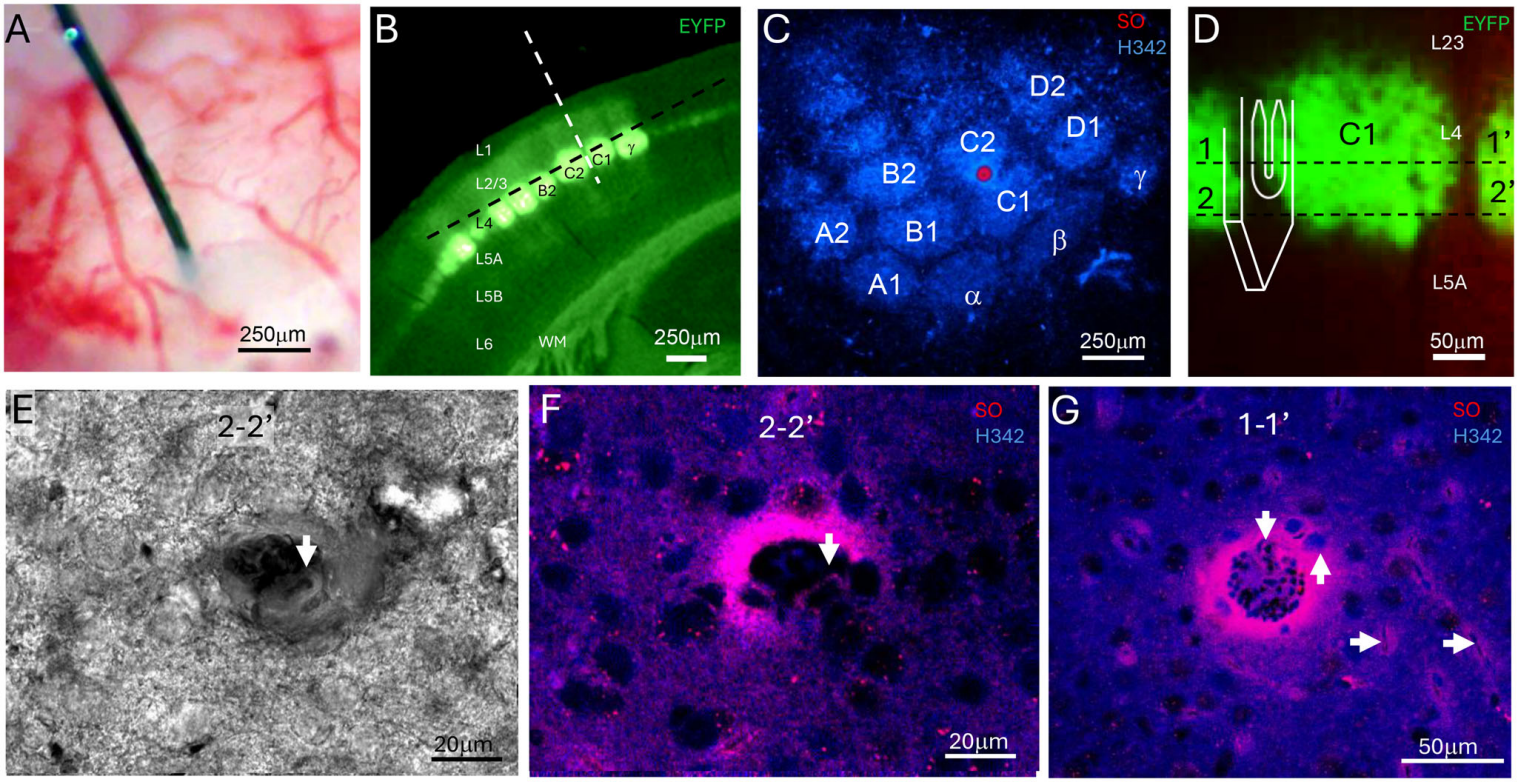
Histological assessment of cell damage during implantation and operation of open‐flow ND probe. (A) Photograph of the probe needle implanted into the mouse SSP‐bfd cortex (2 mm AP, 3.4 mm ML from Bregma, 37° off DV axis). (B) Fluorescence microscope image of the coronal slice taken with a green EYFP filter. Probe position is shown by a white dashed line. The black dashed line corresponds to the tangential section. (C) A confocal fluorescence image of the tangential section showing the barrel field (pseudo‐blue, H342 channel) and the overlaid probe track (pseudo‐red, SO channel). (D) Schematic of the needle sampling area (to scale) overlaid on a fluorescence coronal section showing the C1 barrel in Layer 4. Positions of two tangential slices, 1‐1’ at 400 µm depth, and 2‐2’ at 450 µm depth are shown by black dashed lines. (E) Bright‐field confocal image of the tangential slice 2‐2’. (F) Confocal fluorescence image of the tangential slice (E), taken with red (SO) and blue (H342) channels. The top‐down arrow shows the breached membrane. (G) Confocal fluorescence image of the tangential slice 1‐1’ taken with red (SO) and blue (H342) channels. The top‐down arrow shows the breached membrane. The bottom‐up arrow shows an intact cell with a blue‐stained soma. Horizontal pointed arrows show microvasculature.

To measure local chemical gradients in the brain ECS using implantable chemical probes, it is of paramount importance to avoid extensive tissue damage and minimize the breach of blood–brain barrier (BBB). Both contribute to the intermixing of extracellular chemicals across the damaged area, to contamination of dialysate with intracellular cytosol content that leaks through punctured cell membranes, and further contamination by blood content via breached BBB [32]. Figure 2A shows a silicon ND probe implanted into the center of the barrel field of the primary somatosensory cortex (SSP‐bfd) of an anesthetized mouse (SI). As opposed to MD or PPP probes based on glass capillaries [10, 12] (10^5^ µm^2^ footprint) or on silicon micro‐fabrication [15, 16, 17, 18, 19] (>6000 µm^2^), the small footprint of the ND probe (1750 µm^2^) avoids large surface and penetrating arterioles and venules (Figure 2A) that in the mouse cortex typically are separated by mean distance [33] of 130–150 µm. Histology of a coronal section (Figure 2B) of a Scnn1A‐Cre × Ai32 crossed transgenic mouse with strong expression of EYFP in Layer 4 (SI), shows the targeted implantation position of the ND probe.

To reconstruct the probe position and to characterize the cellular damage imposed by the 180 min chemical sampling at 4nL/min flow rate, 4 µM of Hoechst 33342 (H342) and 250 nM Sytox Orange (SO) staining dyes (SI) are added to the perfusate for staining cells with intact and with compromised membranes, respectively. Confocal fluorescence imaging of a tangential section reveals a barrel field (H342 channel, pseudo‐blue) with clearly identifiable individual barrels (Figure 2C) and the overlaid probe track (SO channel, pseudo‐red). Close inspection of such consecutive confocal scans of individual slices (SI) enables reconstruction of the probe position (Figure 2D) with the sampling area located near the center of Layer 4 between C1 and C2 barrels.

High‐resolution confocal bright field (Figure 2E) and overlaid fluorescence images (Figure 2F) taken at 450 µm depth directly below the sampling area (slice 2‐2’ in Figure 2D) reveal the tissue is mostly affected by shear stress during probe implantation, rather than by open‐flow sampling. The physical scar containing fragments of broken membranes (Figure 2E, top‐down arrow) is circular with an area of 1500 µm^2^. The red staining (Figure 2F, SO channel) does not extend beyond 10 µm from the scar, presumably due to strong uptake into the neuropil with no clearly stained cells within its reach. Blue channel Figure 2F shows black unstained neuron somata decorated by the blue neuropil background, presumably due to autofluorescence rather than H342 staining. The confocal fluorescence image (Figure 2G) of the slice taken at 400 µm depth (slice 1‐1’ in Figure 2D) corresponds to the tissue subjected to prolonged open‐flow sampling. The red channel reveals much stronger neuropil staining extending beyond 20 µm from the scar due to stronger diffusion fluxes. The SO staining appears not only on the front surface close to the open sampling area but also forms a ring extending to the bottom probe surface. However, no clearly stained somata are visible within this ring, indicating minimal membrane damage. Indeed, somata within the nearest 20 µm are stained blue, indicating intact cell membranes (down‐up white arow). Volumetric cell counting for both slices reveals no degradation across the scar region (Figure S1) with neuron cell densities (10^5^ neurons/mm^3^) commensurate with literature data for layer 4 of a barrel cortex [34, 35].

Overall, these observations demonstrate that cell damage does not extend beyond the physical footprint of the probe and corresponds to a total number of membrane‐compromised neuron somata estimated in the 10 to 20 range within the volume of 1.75 × 10^−4 ^mm [3] along 100 µm length of the sampling area. Density of non‐neuronal glial cells [36] and, specifically, astrocytes [37] are an order of magnitude lower (10^4^ neurons/mm^3^) resulting in an upper estimate of compromised glial cells fewer than 10. Notably, the ND probe footprint is of the order of the distance between the smallest sub‐surface micro‐vasculature capillaries (horizontal pointed arrows in Figure 2G), ranging between 10 to 30 µm in the mouse cortex [38], further minimizing dialysate contamination from the BBB breach.

### 2.3 Diffusive Transport and Clearance Rate of Exogenous Molecules in the Mouse Cortex In Vivo

**FIGURE 3:**
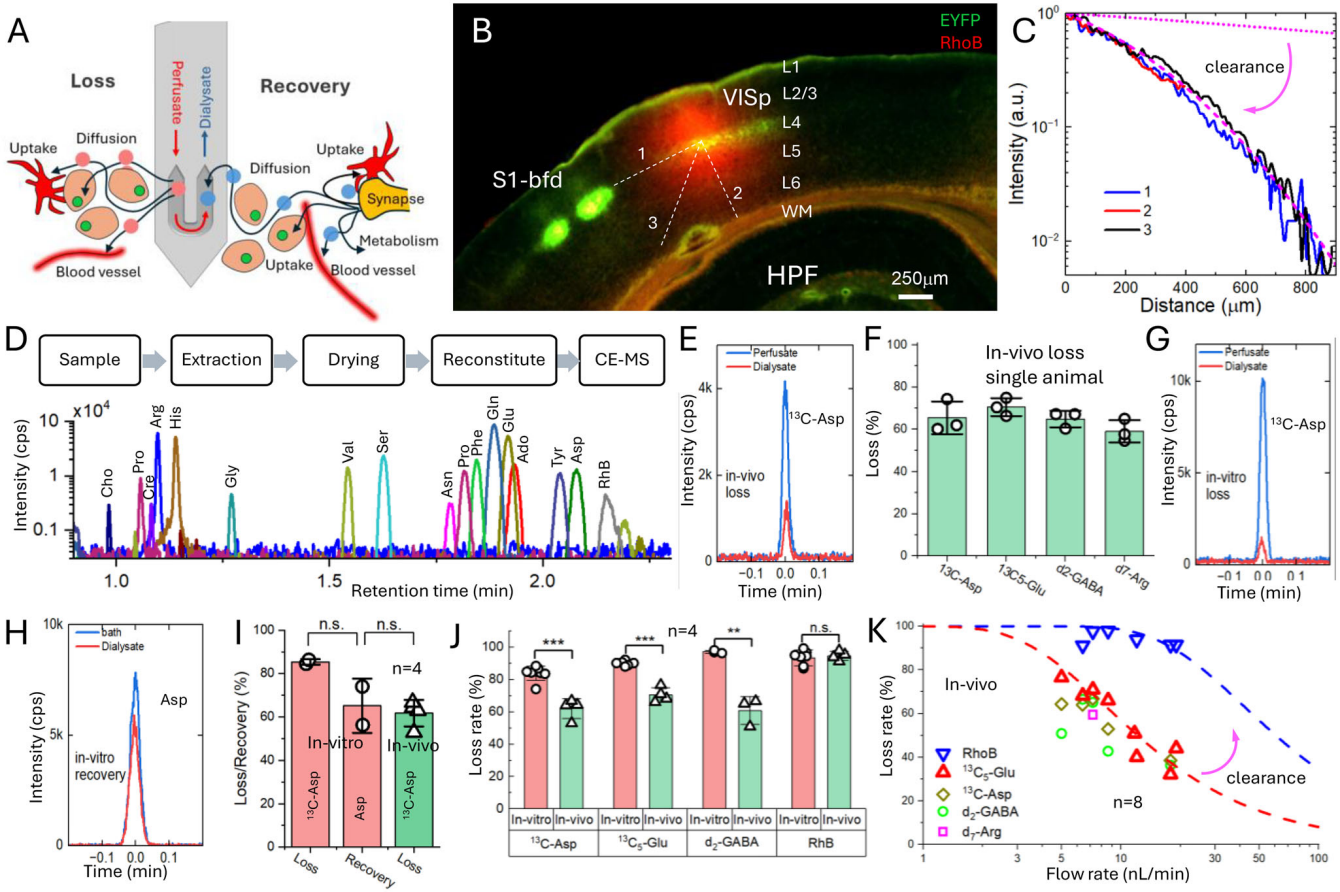
Mass spectrometry determination of in vivo recovery, loss, and clearance rates. (A) Schematics of different pathways for neurotransmitters and metabolites that contribute to in vivo recovery and loss. (B) Fluorescence microscope image of the coronal slice combined from green (EYFP) and red (SO) channels. White dashed lines are used for intensity analysis. (C) Intensity profile of RhB fluorescence measured along different directions in (C). Dashed and dotted lines are fits to the diffusion equation (SI). (D) Inset: Schematic of the CE‐MS sample preparation and workflow. Electropherograms of multiple neurotransmitters and metabolites detected with CE‐MS from an in vivo sample in (B). Semi‐log scale. (E) Electropherograms of perfusate and in vivo collected dialysate showing loss of 13C‐Asp. (F) Loss rates for several isotope‐labeled standards measured in vivo for a single animal. Circles correspond to three technical replicas. (G) Electropherograms showing in vitro loss of 13C‐Asp. (H) Electropherograms showing in vitro recovery of 13C‐Asp. (I) Comparison of in vitro loss (13C‐Asp), in vitro recovery (Asp), and in vivo loss (13C‐Asp) for all technical (circles, *n* = 2) and biological (triangles, *n* = 4) replicas sampled at 7nL/min. (J) Comparison of in vitro and in vivo loss for technical (circles, *n* = 7) and biological (triangles, *n* = 4) replicas for isotope labeled standards and RhB sampled at 7nL/min. (K) In vivo loss rate for isotope‐labeled standards and for RhB as a function of flow rate. Dashed lines are fits to Equation (1) with different effective resistances.

As opposed to diffusion transport in vitro, neuroactive analytes in vivo diffuse in the ECS [39] within the volume restricted to a fraction α of just 20% and constituted of nanoscale interstitial channels and the extracellular matrix scaffold with tortuosity λ in the range 1.4 to 1.6 (Figure 3A). Moreover, while travelling in the ECS, analytes undergo complex interactions with the tissue, including clearance via binding to membrane receptors, uptake into astroglia and vasculature, as well as metabolic degradation (Figure 3A). In the steady‐state model [13, 14] of Equation (1), assumpting radial diffusion into uniform tissue and a linear clearance rate, the loss of analyte from the ECS to these active processes is described by a first‐order loss rate constant *k_ECS_*. Therefore, the analyte transport in the brain can be described [39] by a modified diffusion equation:

(2) $$\partial C/\partial t=D/\lambda^{2}\nabla^{2}C+Q/\alpha -k_{ECS}C$$

The first term defines diffusion in the restricted ECS space, and it tends to equilibrate chemical gradients. The second term (sources) *Q*/α that defines analyte secretion with flux *Q*, and the third term (sinks) that defines analyte clearance both contribute to the establishment and maintenance of strong chemical gradients crucial for paracrine cell‐to‐cell communications [5]. In particular, the concentration profile near the probe surface becomes non‐uniform at equilibrium with a characteristic penetration depth $\Gamma \;=\sqrt{D^{*}/k_{ECS}}$. If the clearance is fast (case of SO and H342 in Figure 2), Γ shrinks to just a few tens of microns (Figure 2G) accounted for [13] in Equation (1) by the tissue mass transport resistance *R_ECS_*.

To visualize and quantify diffusive transport in the mouse cortex in vivo, we added to the perfusate 10 µM of the exogeneous dye Rhodamine B (RhB) (Figure 3B) to the perfusate. RhB is a small molecule (479 Da), and its cation usually appears around 443 m/z in ESI or LC‐MS spectra, in the middle of our target small molecule analytes of interest. It is a high quantum yield fluorescence marker with emission distinct from other compounds we use in histological assessments. The intensity of RhB fluorescence measured (SI) both along and perpendicular to the cortical layers (Figure 3C) is virtually identical, indicating a high degree of homogeneity and diffusion isotropy of a primary cortex. The characteristic decay of intensity is well described (SI) by the error‐function solution to a diffusion equation (dashed line in Figure 3C) with an effective diffusion coefficient D* of 6 × 10^−8^ cm^2^/s. This is 100 times smaller than diffusion of RhB in water [40] corrected to 37°C brain temperature (*D* of 6 × 10^−6^ cm^2^/s). Accounting for the ECS tortuosity λ of at most 1.57 reported for the SSP cortex of adult mice [39], the effective D* = *D*/λ^2^  is reduced by only 2.5× to 2.4 × 10^−6^ cm^2^/s (dotted line in Figure 3C). The remaining reduction is then defined by the active clearance with *k_ECS_* rate of the order of 10^−3^/s corresponding to a penetration depth Γ of several hundred microns as observed in Figure 3B,C. With such a modest clearance rate defined by the transmembrane diffusion [41], it serves as a good reference for identifying the rate‐limiting barrier for diffusion of our target endogenous molecules.

### 2.4 Determination of In Vivo Recovery, Loss, and Clearance Rates With CE‐MS

To measure chemical content of collected sub‐µL dialysate samples, we adopted capillary electrophoresis mass spectrometry (CE‐MS) that is compatible with small sample volumes, provides high separation efficiency [42] to resolve complex mixtures of brain neurochemicals, and is well suited for biological samples with high saline content [43].

The preparation workflow (inset in Figure 3D) consists of sample extraction from the probe, followed by vacuum drying to remove residual oil used during ND probe operation, and reconstituting the residue to 5 µL prior to injection to a single‐chip CE electro‐spray‐ionization (ESI) system directly attached to a Q‐TOF mass spectrometer (Supporting Information, SI). To identify collected analytes and estimate their concentration, a reference standard solution containing 18 major small‐molecule neurochemicals, including 7 neuroactive neurotransmitters and 11 other metabolites, was prepared in artificial cerebrospinal fluid (aCSF) and used to obtain calibration curves and limits of detection (LOD). A typical electropherogram (Figure 3D) of a dialysate sample collected in vivo for 2 h at 7nL/min flow rate (SI) from the location in Figure 3B reveals multiple peaks appearing at different retention times. Retention times and intensities (area under the peak) of each peak are compared to electropherograms of a reference standard solution (SI, Figure S2A) with known concentration, migration times, and mass to charge ratios.

To measure reliably local chemical gradients in the brain, precise calibration of in vivo sampling experiments from different brain areas and between different animals is vital. Standard methods for in vivo *RR* calibration [44] require a number of additional sampling experiments that are difficult to perform in situ without further perturbing the tissue. Instead, we adopted an alternative method [45] based on measurements of loss rate (*LR*) of endogenous stable isotope labeled standards (SILS) collected in situ during the same sampling experiment. For that purpose, 2 µM SILS of two major neurotransmitters (d_2_‐GABA, ^13^C_5_‐Glu) and two metabolites (^13^C‐Asp, d_7_‐Arg) were added into the perfusate (Figure S2C). These 4 analytes cover the range of polarities, molecular weights, diffusion coefficients, and tissue clearance rates that are of interest for the current study and their addition to the tissue at these relatively low concentrations and with ultra‐low nL/min flow rates used in our experiments should not significantly affect concentration levels of their endogenous forms [45] in the brain.

A pair of typical in vivo electropherograms, for example for ^13^C‐Asp SILS (Figure 3E), compares the peak areas of dialysate and perfusate, yieding an *LR* of 69.5%. When measured for all four SILS (Figure S2D), with three technical replicates each (Figure 3F), all mean *LR* values are above 60%. To estimate contributions of various resistances in Equation (1) to the observed in vivo *LR*, in vitro measurements of *LR* (Figure 3G) and *RR* (Figure 3H) were performed (SI) and compared to in vivo results (Figure 3I). To average out the biological variables, the in vivo measurements were performed on 4 animals (Table S1) producing *LR* value for ^13^C‐Asp of 61.8% ± 6.1% (mean ± SD). This is lower than the in vitro *RR* of 74.9% ± 13.5%, and the in vitro *LR* of 83.9% ± 4.7%, but differences are not statistically significant (Figure 3I). This observation is similar to measured ratios of in vivo and in vitro *RR* in traditional low‐flow MD [9]. However, for MD, the major rate‐limiting factor [13, 14] for analyte recovery is *R_ECS_* for most analytes.

In Equation (1), the large resistance of the dialysis membrane *R_m_* under typical conditions significantly exceeds the tissue resistance for actively cleared neurochemicals with large enough *k_ECS_*. Our open‐flow ND design operates in a drastically different regime (*R_m_* =  0), where *R_ECS_* and *R_d_* instead become rate‐limiting factors.

The population‐averaged in vivo *LR* for all SILS is significantly lower than the corresponding in vitro *LR* (Figure 3J) that are all above 90%, indicating that the rate‐limiting barrier for diffusion is related to *R_ECS_* rather than *R_d_*. In contrast, the in vivo and in vitro *LR* for RhB are all above 90%, showing lowered diffusion barrier presumably due to its fast clearance. Increase of the flow rate (Figure 3K) results in decrease in the *LR* for all SILS measured across eight animals as expected from Equation (1). When less scattered ^13^C_5_‐Glu data (red triangles) are fitted with Equation (1), the resulting effective resistance is 3 × 10^6^ s/µm. The upper limit of *R_d_* of 6 × 10^5^ s/µm can be estimated from fitting the RhB data (blue triangles), assuming that its relatively high *k_ECS_* results in negligible *R_ECS_*. Then *R_ECS_* for all measured SILS can be estimated as 2.4 × 10^6^ s/µm in assumption of their clearance rate *k_ECS_* being smaller than 10^−3^/s.

### 2.5 Measurements of In Vivo Chemical Gradients Along BFD‐LL Axis

**FIGURE 4:**
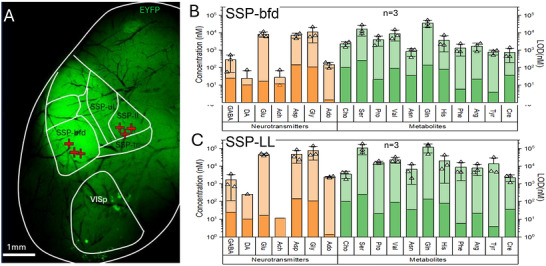
Measurements of in vivo chemical concentrations of neurotransmitters and metabolites in SSP‐BFD and SSP‐LL areas. (A) Top‐down fluorescence microscope image of the mouse brain taken with green (EYFP) filter. Crosses correspond to identified probe tracks for six animals. (B) Mean concentration of neurotransmitters (light brown bars) and metabolites (light green) for three animals (triangles) with sampling from the SSP‐bfd area. Dark brown and dark green bars correspond to measured limits of detection (LOD, right vertical axis). Bars show mean value, whiskers correspond to 1SD. (C) Mean concentration of neurotransmitters (light brown bars) and metabolites (light green) for three animals (triangles) with sampling from the SSP‐ll area. Dark brown and dark green bars correspond to measured limits of detection (LOD, right vertical axis). Bars show mean value, whiskers correspond to 1SD.

To measure basal concentrations of neurochemicals in vivo, the dialysate samples were collected (SI) from 3 animals (Table S1) with the ND probe targeting SSP‐bfd (Figure 4A). We traced the position of the open sampling area for each experiment from histological analysis as shown in Figure 2B,D by estimating its position from the center of RhB fluorescence. For all the animals we studied, the estimated cortical depth was 400 ± 100 µm. Absolute concentrations were determined (SI) using separately acquired calibration curves and corrected for in vivo recovery using the in vivo *LR* of ^13^C_5_‐Glu added to the perfusate. The concentration levels of 7 neurotransmitters and 11 metabolites measured in the SSP‐bfd (Figure 4B) cover a large dynamic range between 42 µM for Gln down to 43 nM for Ach (SI Table S2).

Utilizing the high spatial resolution of the ND probe, in vivo sampling was performed in additional cohort of three mice (Table S1) targeting an adjacent lower limb primary cortex region SSP‐ll (Figure 4A). Corresponding concentrations (Table S2) exhibit similar trends, however, are generally higher than in SSP‐bfd (Figure 4C).

**FIGURE 5:**
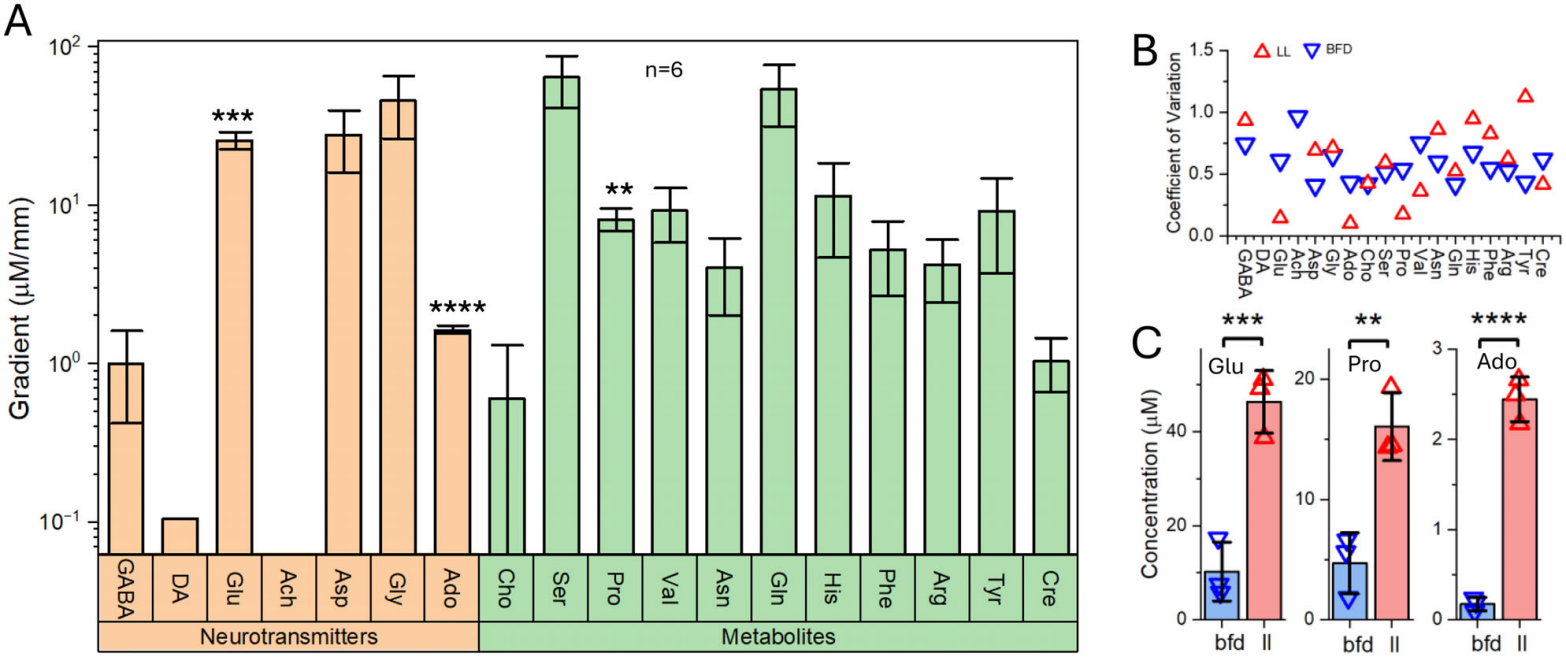
Measurements of in vivo chemical gradients along BFD‐LL axis. (A) Concentration gradient between SSP‐bfd and SSP‐ll areas. The bar represents the mean, whiskers correspond to pooled SD. Corresponding *p* values shown by stars. (B) Coefficient of variation of measured concentrations in (B) for SSP‐bfd (down blue triangles, *n* = 3 animals) and SSP‐ll (top red triangles, *n* = 3 animals). (C) Comparison of concentrations of several neurochemicals measured in SSP‐bfd (down blue triangles, *n* = 3) and SSP‐ll (top red triangles, *n* = 3). Corresponding *p* values are shown by stars.

The calculated concentration gradients (Figure 5A) are overall positive, varying between 100 nM/mm for DA up to 64.5 ± 0.7 µM/mm for Ser. While numerically large, not all of these values reach statistical significance. Note that the reported mean concentrations (Figure 4B,C) represent values calculated over 3 mice with three technical replicas each, with biological variables producing by far the largest contribution to the coefficients of variation (CV) (Figure 5B). The largest CVs correspond to GABA and Ach with the lowest ratio of their mean value to the LOD (dark colors in Figure 4B,C), while the smallest CV observed for Ado is dictated by its LOD as small as 1.45 nM. In fact, only Glu, Pro, and Ado produce statistically significant concentration differences of 25.8 ± 3.3 µM/mm, 8.1 ± 1.4 µM/mm, and 1.6 ± 0.1 µM/mm with *p* values (two‐sample *t*‐test) of 1.8 × 10^−3^, 1.0 × 10^−4^, and 5.9 × 10^−3^, respectively (Figure 5C).

## 3 Conclusions

Two major advantages of the open‐flow ND probe in minimizing tissue damage (within 70 × 25 µm^2^ footprint), and in measuring highly localized chemical content (defined by *S* of 4200 µm^2^), may be largely responsible for generally higher measured concentrations of neurotransmitters and metabolites (Table S2, Figure 4B,C) than previously reported [10, 46, 47, 48, 49]. For example, highest measured extracellular Glu (eGlu) concentration (46 µM in SSP‐ll) is 80‐times higher than 0.6 µM measured with a standard MD probe [48] (*S* of 10^8^ µm^2^), and 2‐times higher than 26 µM measured with more localized low‐flow PPP [10]. It is, however, very close to 44 µM measured with electrochemical biosensor [50] with *S* of 7500µm^2^. This is consistent with the general trend observed in the literature that smaller footprint electrochemical sensors yield eGlu concentrations within a range (4 to 44 µM) one order of magnitude higher than measured with traditional large‐area MD (0.3 to 3 µM). It has been suggested [25] that different techniques are possibly measuring distinct extracellular compartments with varying eGlu concentration, just with different biases to synaptic‐originated and non‐synaptic glia‐originated pools.

Our results indicate that strong chemical gradients of most measured analytes build up at the mm‐scale distances separating SSP‐bfd and SSP‐ll cortical areas. In the case of eGlu, the gradient is as large as 25.8 ± 3.3 µM (Figure 4E) across a distance of 1 mm, which is 500‐times larger than extrasynaptic overspill of neurotransmitters over less than 2 µm distances from the synapse [27, 51, 52]. Notably, these areas possess very similar ECS diffusion transport characteristics (first term in Equation 2) as attested by observation of homogeneous and isotropic spread of exogenous RhB dye (Figure 3BC). Therefore, the competition of eGlu release of non‐synaptic glial origin [53] (second term in Equation 2) and uptake by astrocyte transporters [30] (third term in Equation 2) is likely responsible for formation and maintenance of these highly non‐uniform ECS chemical gradients.

Other neurotransmitters and metabolites also exhibit substantial concentration differences (Table S2) consistent with the role of astroglia in regulation of ECS neurochemistry. For example, elevated level of Gln correlated with higher eGlu in SSP‐ll is consistent with astrocyte‐neuron Glu–Gln cycle regulated by astrocyte membrane transporter proteins [54]. An increase of the extracellular levels of Ser, where release and uptake are tightly regulated by astrocyte‐neuron interactions [55, 56], although not reaching statistical significance, produces gradients above 60 µM/mm. It is interesting to speculate whether the Ser detected is L‐Ser or D‐Ser, as the latter interacts with the Gly binding site on the N‐methyl‐D‐aspartate (NMDA) receptor [55, 57] and thus impacts Glu signaling. Our current system cannot resolve D‐Ser for L‐Ser but such measurements are possible with alternative CE‐MS separation schemes. We also see statistically significant changes (Figure 5C) in Pro (8.1 ± 1.4 µM/mm gradient), which is metabolized via astrocyte‐neuron lactate shuttle [58]. In addition, the statistically significant gradient for Ado (1.6 ± 0.1 µM/mm) (Figure 5C) also likely involves astrocyte and microglia regulation [59] via conversion from extracellular ATP.

One of the disadvantages of the current generation of the probes is that concentration measurements are averaged over the whole duration of the sampling time as analytes are accumulated in the same location at the probe outlet (Figure 1). We are, however, creating a variant of this probe that can provide temporal chemical measurements with sub‐second time resolution [21]. For that purpose, the analytes are segmented on‐chip with oil plugs into a sequence of isolated pL‐scale droplets [60, 61] and then analyzed one‐by‐one by mass‐spectrometry [22, 23, 24].

Another potential problem is probe clogging facilitated by ultraslow flows. We observed, however, that it is not induced by clogging of the open sampling area that remains operational for several hours. Clogging is mostly defined by obstruction of the microfluidic channels themselves owing to their reduced cross‐section. We are mitigating that by cleaning the perfusates from particles and by adding special particle‐trapping devices at the inlet ports.

Taken together, our findings indicate that in otherwise diffusion‐homogeneous cortical areas, strong chemical gradients are maintained in the ECS in vivo at the mm‐scale that likely are controlled by glial‐neuron interactions [26, 27, 28] that may strongly influence physiological and pathophysiological modulation of neural computations [29, 30]. Thus, our approach casts an unbiased net to find known and unknown neurochemicals that have unexpected local concentration changes, allowing for directed follow‐up studies important for understanding the astrocyte‐neuron interactions in health and disease.

## Conflicts of Interest

The authors declare no conflicts of interest.

## Supporting information




**Supporting File**: It contains experimental details, two supplemental figures, and two tables.

## Data Availability

The data that support the findings of this study are available from the corresponding author upon reasonable request.
