# Supplementary material for: Local Chemical Gradients in a Mammalian Cortex Measured In Vivo With a Silicon Nanodialysis Mass Spectrometry Platform
Source: Angew Chem Int Ed Engl. 2026 May 6;65(26):e3609448. doi: 10.1002/anie.3609448 (PMC13285464; doi:10.1002/anie.3609448)
Supplement: Supplementary file 1 — Supporting File: It contains experimental details, two supplemental figures, and two tables. [file ANIE-65-e3609448-s001.pdf]

Supporting information

Local chemical gradients in a mammalian cortex measured in-vivo with silicon nanodialysis mass spectrometry platform.

Weihua Shi[1†], Keyin Li[2†], Yu Ding[1], Alex G. Armstrong[2], Jonathan V. Sweedler[3,4,6*], and Yurii Vlasov[1, 2, 4, 5, 6*]

[1] Electrical and Computer Engineering Department,

[2] Neuroscience Program,

[3] Chemistry Department,

[4] Beckman Institute for Advanced Science and Technology,

[5] Carle Illinois College of Medicine

University of Illinois Urbana Champaign, Urbana, IL 61801.

[6] Chan Zuckerberg Biohub, Chicago, Illinois 60642.

*Corresponding authors: Jonathan Sweedler. Email: jsweedle@illinois.edu, Yurii Vlasov. Email: yvlasov@illinois.edu.

† These authors contributed equally to this work

WILEY-VCH

Supporting Information

Probe fabrication and packaging. The details of the microfabrication for channel-embedded silicon probes have been reported in our previous work on silicon microfluidic platforms. In brief, the ND probes are fabricated using a silicon-on-insulator (SOI, UltraSil) wafer with a 425 µm handle thickness, a 1µm buried oxide (BOX) layer, and a 20 µm device layer. First, a 300nm of SiNx is deposited to serve as a hardmask for subsequent etching of the Si device layer. Rows of perforations in SiN are defined by photolithography followed by reactive ion etching, leaving perforations of 1µm diameter exposing the Si device layer below. Next, the exposed Si is isotropically etched, forming a continuous microfluidic channels buried underneath the SiNx hardmask. The perforations in the SiN hardmask layer are then sealed by deposition of 3µm low stress SiN. Individual chips and the probe needles are released with silicon deep reactive ion etching from the top and backside handle layers. To interface with external microfluidic pumps, the 700 µm ID silica capillaries (TSP700850, Polymicro) are attached to inlet and outlet ports of the chip and the final 3D printed package is sealed with UV resin (**Fig.1A**).

Chemicals. Artificial cerebral spinal fluid (aCSF) was purchased from Tocris Bioscience. 1-Octanol was purchased from Thermo Scientific Chemicals. To dye the 1-Ocatnol for easy distinction from the aqueous phase, β-Carotene from Sigma Aldrich (St. Lousi, MO) was used. Neurotransmitter and amino acid standards were purchased from Sigma Aldrich (St. Louis, MO), and stable isotope-labeled standards were purchased from Cambridge Isotope Laboratories, Inc (Andover, MA). All stock solutions were prepared with LC-MS grade water. To stain cells with intact and with compromised membranes respectively, Hoechst 33342 and Sytox Orange staining dyes from Sigma Aldrich (St. Louis, MO) were used.

Sample Preparation and CE-MS Analysis. The push and pull channels of the probe are connected to pressure pumps (Fluigent Push-Pull) via attached capillaries and Teflon tubing for precise control of the flow rates (**Fig.1A**). The perfusate capillary is loaded with SILS and dyes in aCSF when specified. To precisely measure volume of collected dialysate sample, the dialysate capillary is filled with inert 1-Octanol oil.

Following sampling, the probe is retracted from the implantation site, and a Hamilton glass syringe (Hamilton 701N) is utilized to extract the dialysate. 5 µL of diluent (50% methanol) was added into the dialysate and the entire aqueous phase was transferred into a microcentrifuge tube. Any residual octanol was removed by drying the sample in a vacuum concentrator (Genevac, Ipswich, UK). The dried samples were stored at -80°C until CE-MS analysis.

CE-MS analysis was performed using a ZipChip microfluidic system (908 Devices, Boston, MA) attached to a Q-TOF mass spectrometer (Bruker maXis 4G, Bellerica, MA). The mass spectrometer was calibrated regularly using 10 mM sodium formate for mass range of m/z 70 – 1000. High speed chips (908 Devices, Part# 810-00195) and metabolite background electrolyte (908 Devices, Metabolites Reagent Kit Part# 810-00033) were used for all sample analysis. Samples were reconstituted in 5 µL of Metabolite Diluent on the day of analysis and were loaded manually into the sample well. CE separations were performed with a field strength of 1000 V/cm and an injection volume of 5 nL. Run times were 3 min. Triplicate injections (technical replicas) were performed for each sample, except for two in-vivo dialysate samples where only a single injection was possible due to limited collected volume.

Calibration curves for each analyte were constructed with the following concentrations: 40, 100, 300, 600, 1200, 2000 nM. Linearity of calibration curves was estimated with R^2 coefficient 0.9904±0.0055 with minimal 0.98083 for Ser and maximum 0.9977 for Ado.

MS Data Analysis. Data analysis was performed using Compass DataAnalysis 4.4 (Bruker Corp., Bellerica, MA). Peaks were identified by matching migration time and mass to charge ratio to analytical standards. Peaks were integrated using the Find Compound – Chromatogram function in DataAnalysis and inspected manually. Statistical analysis was performed using OriginPro 2024 (OriginLab Corp., Northampton, MA).

In-Vitro Recovery Rate Characterization. To evaluate the in-vitro recovery rates, the ND probe was used to sample from a bath solution containing γ-aminobutyric acid (GABA), adenosine (Ado), glutamate (Glu), glycine (Gly) and aspartate (Asp) with 2 µM concentration each. The bath solution was maintained at 38°C and stirred continuously throughout the experiment. The push-pull flow rate was controlled at 10 nL/min, and dialysate was collected over a 1-hour period into the outlet silica capillary. Two experimental replicates were performed for in-vitro sampling experiment using separate ND probes under the same flow rate. Dialysate and bath samples were analyzed by capillary electrophoresis mass spectrometry (CE-MS), and relative recovery rate was calculated as: Recovery (%) = $(\text{Peak Area}_{\text{dialysate}}/\text{Peak Area}_{\text{bath}}) \times 100\%$.

In-Vitro Loss Rate Characterization. To assess in-vitro loss rates, the perfusate was supplemented with 10 µM of rhodamine B (rhoB), and 2 µM of d_2-GABA, $^{13}C_5$-Glu, ^{13}C-Asp, and d_7-Arg during the in vitro sampling experiment described above. The dialysate and perfusate were analyzed by CE-MS and relative loss rate was calculated as: Loss (%) = $(1 - \text{Peak Area}_{\text{dialysate}}/\text{Peak Area}_{\text{perfusate}}) \times 100\%$.

Animals. This study is based on data from 8 mice of both sexes aged between P57 and P234 (**Table.S1**). To ease identification of principal barrel and enable optogenetic tagging, the transgenic Scnn1a-TG3-Cre (Jackson:009613) line was crossed with

the Ai32 line (Jackson: 012569), producing strong co-expression of the enhanced yellow fluorescent protein (EYFP) in layer 4 excitatory neurons in the primary somatosensory cortex (**Fig.2B, 4A**).

Surgical Procedures. All procedures were in accordance with protocols approved by the UIUC Institutional Animal Care and Use Committee. Animals were kept on a reverse 12-hour light/dark cycle in individual cages equipped with activity wheels (K3250 Fast-Trac, Bio-Serv) to encourage active behavior in-between experimental sessions. All experimental sessions were performed during the dark phase. Surgical procedures were carried out aseptically while animals are anesthetized with isoflurane (2-4% in oxygen) before being injected subcutaneously with 5mg/kg carprofen. Custom-built titanium headbar (10mm x 2 mm) was attached to the animal's skull using VetBond (3M, USA) to hold the animal's head. Orientation of the headbar is carefully adjusted with respect to Bregma and Lambda points on the animal skull to provide a repeatable coordinate system for ND probe insertion. The central open area of the headbar (3mm x 2mm) exposes the skull directly above the primary somatosensory cortex (SSP-bfd and SSP-ll) for in-vivo visualization, targeted craniotomy, and precise probe insertion. This area is covered in optical grade clear cement for in-vivo fluorescence imaging. Beginning the day following the surgery, subcutaneous Carprofen (5mg/kg) is administered for two days to reduce inflammation.

After complete recovery for 4 days, on the morning of the final terminal experimental session, mice are briefly anesthetized with isoflurane (2-4% in oxygen) and are placed in a head-fixation surgery apparatus for targeted ND probe insertion. Anesthesia was maintained at 1–2% isoflurane level throughout the procedures. Animals were kept on a heat pad to maintain body temperature, and ophthalmic ointment was applied to protect the eyes from desiccation.

Using a microdrill with 0.002" burrs (Fine Science Tools, Canada), the optical cement and the skull are carefully thinned while the surface is cooled down using sterile artificial cerebrospinal fluid (ACSF). A small craniotomy of < 300μm diameter is opened while the dura remained intact. The craniotomy site is then imaged both in a white light and with fluorescence filters to visualize a barrel field and a vasculature pattern and to produce a detailed map for probe implantation (**Fig.4A**).

In-Vivo Sampling. ND probes were mounted to a custom 3D-printed holder and attached to a steel rod held by a 3D robotic arm (Sensapex) at 38 degrees around the animal head AP axis to ensure shank was perpendicular to cortical layers in the C2 barrel column of the SSP-bfd (**Fig.2B**).

The push and pull channels of the probe are connected to pressure pumps (Fluigent Push-Pull) via attached capillaries and Teflon tubing for precise control of the flow rates (**Fig.1A**). The perfusate capillary is loaded with SILS and dyes. To prevent the loss of collected dialysate sample and measure its volume precisely, the dialysate capillary is initially filled with inert 1-octanol oil, dyed with 2mM of β-Carotene.

After dura penetration, the probe is implanted at a rate of 100 μm/min until the target depth of 500 μm is reached. During implantation a constant pressure of +100 mbar is supplied to both perfusate and dialysate channels to provide 5nL/min backflush flow and prevent tissues from clogging the sampling area. Once the probe reached the target depth, the flow configuration was switched to a push-pull mode with a constant flow rate of 7nL/min.

During sampling the dialysate is accumulated into the capillary attached to the probe outlet. Slow movement of the meniscus along the outlet capillary between oil and accumulating dialysate is observed with attached microscope (Supereyes, Shenzhen) to estimate the average flow rate. After completion of experiment a single dialysate fraction (0.65-1.2 μL) was collected over a 1.5–2 hour period from each animal. Three biological samples were collected from each SSP subregion, barrel field (SSP-bfd) and lower limb (SSP-ll) (**Table S1**).

In-Vivo Loss Rates and Basal Concentrations. The perfusate for in-vivo sampling experiments contained 10 μM rhodamine B and 2 μM of d_2-GABA, $^{13}C_5$-Glu, and ^{13}C-Asp as internal standards for calculation of in vivo loss rates. CE-MS was used to analyze both perfusate and dialysate. The in-vivo loss rate was calculated as: Loss (%) = (1 – Peak $Area_{dialysate}$/Peak $Area_{perfusate}$) × 100%. The loss rate of $^{13}C_5$-Glu in each experiment was used to correct the basal concentrations of all measured analytes.

Histology and confocal microscopy.
Immediately after the end of final terminal experiment, mice were deeply anesthetized with 5% isoflurane then drop-fixed in 4% paraformaldehyde to enable vasculature to be visible. The brains after fixture were imaged with top-down fluorescence microscopy with camera-equipped Olympus MVX10 epi-fluorescence microscope (**Fig.4A**) that enables matching of postmortem vasculature patterns and SSP fields to identify the entry point of the probe with the cortical field map. The brains were then sectioned into 50-μm-thick coronal sections (VT1200S, Leica). Strong EYFP expression in Layer 4 clearly identifies individual barrels (**Fig.2B**).
For identification of cell damage a tangential sectioning is performed (**Fig.2C**) and slices were imaged with confocal laser-scanning microscope (Leica SP8) with 0.35μm step resolution (**Fig.2E,F,G**). Images of individual slices are assigned pseudo-colors using Adobe Photoshop (Adobe Suite) photo-filter option. Cells are identified as black ROI on blue or red stained neuropil background, and their position and density is measured across all scanned slices to provide the volumetric cell density around the probe scar (**Fig.S1**).

Diffusion coefficient fitting. Fluorescence intensity of RhB fluorescence in coronal slices (**Fig.3B**) is measured both along and perpendicular to the cortical layers (**Fig.3C**). The resulting traces are fitted in OriginPro with erfc solution for a diffusion transport from a point source with constant concentration $C(x) = A \cdot erfc\left(x/2\sqrt{D^{*}t}\right)$, where t is the sampling time, D^{*} is effective diffusion coefficient.

Table S1. List of animals used in this work with reference to figures.

Animal ID	Genotype	Sex	Age (P)	Ctx area	Weight (g)	Figures
2001	Ai32 x Scnn1a (+)	F	105	V1	21	3BCDFK, 4A
2013	Ai32 x Cre (+)	M	53	SSP-bfd	25	3GHIJK
2014	Ai32 x Scnn1a (+)	F	62	SSP-bfd	16	3K,4B,5ABC
110151	Ai32 x Cre (+)	F	57	SSP-bfd	18	3IJK. 4B,5ABC
110157	Ai32 X Cre(-)	M	146	SSP-bfd	26	2ACEFG, 3IJK, 4B,5ABC
578	Ai32 x Scnn1a (+)	F	98	SSP_LL	18	3IJK, 4C,5ABC
2027	Ai32 x Scnn1a (+)	M	151	SSP_LL	24	3K, 4C,5ABC
2030	Ai32 x Scnn1a (+)	M	234	SSP_LL	48	2ABD,3K, 4C, 5ABC

Table S2. Concentration of various neurotransmitters (top) and metabolites (bottom) in the SSP-bfd (left) and SSP-ll (right) cortical areas. Empty positions correspond to values below LOD.

Ctx area	SSP-bfd			Mean	S.D.	SSP-ll			Mean	S.D.	**Gradient**	S.D.	**LOD**	R^2
Anm ID	2014	110151	110157	(nM)		578	2027	2030	(nM)		(μM/mm)		(nM)	
	Neurotransmitters					Neurotransmitters								
GABA	421.6	54.9	531.5	336.0	249.6	695.2	3,595.2	904.3	1,731.6	1,617.3	1.0	0.6	25.2	0.99478
DA	109.5			109.5			256.3		256.3		0.1		10.5	0.99694
Glu	17,359	5,800	7,523	10,227	6,236	51,094	49,160	38,692	46,315	6,672	25.8	3.3	17.0	0.98164
Ach	14.0		73.9	44.0	42.3								11.9	0.99561
Asp	11,242	4,594	9,081	8,306	3,391	84,541	23,385	33,854	47,260	32,708	27.8	11.7	148.0	0.98886
Gly	10,274	5,737	21,506	12,505	8,118	139,859	47,553	43,069	76,827	54,633	45.9	19.7	110.7	0.98651
Ado	238.2	193.0	91.2	174.1	75.3	2,661.9	2,495.3	2,174.9	2,444.0	247.5	1.6	0.1	1.4	0.9971
	Metabolites					Metabolites								
Cho	3,757	1,479	2,953	2,730	1,156	1,817	4,509	4,405	3,577	1,525	0.6	0.7	104.1	0.98777
Ser	18,438	9,043	28,000	18,494	9,479	181,971	59,291	85,279	108,847	64,647	64.5	23.3	264.3	0.98083
Pro	5,638	1,825	6,624	4,695	2,534	19,327	14,363	14,502	16,064	2,827	8.1	1.4	21.6	0.98795
Val	10,167	4,584	15,028	9,926	5,227	32,414	17,416	18,817	22,882	8,285	9.3	3.5	94.3	0.98481
Asn	1,599	386	1,063	1,016	608	13,140	2,071	4,883	6,698	5,753	4.1	2.1	35.4	0.99017
Gln	50,144	21,776	53,361	41,760	17,382	47,903	164,274	141,485	117,887	61,670	54.4	22.9	140.7	0.98426
His	2,928	2,110	7,254	4,097	2,764	42,070	11,595	6,908	20,191	19,092	11.5	6.9	80.9	0.98896
Phe	1,207	855	2,408	1,490	814	17,259	4,576	4,679	8,838	7,293	5.2	2.6	5.9	0.9977
Arg	2,798	806	2,445	2,016	1,063	13,554	5,911	4,320	7,928	4,936	4.2	1.8	21.8	0.9938
Tyr	1,297	501	970	922	400	31,696	4,538	5,187	13,807	15,496	9.2	5.5	4.0	0.99668
Cre	636	434	1,406	825	513	1,185	2,915	2,745	2,282	954	1.0	0.4	36.9	0.99338

WILEY-VCH

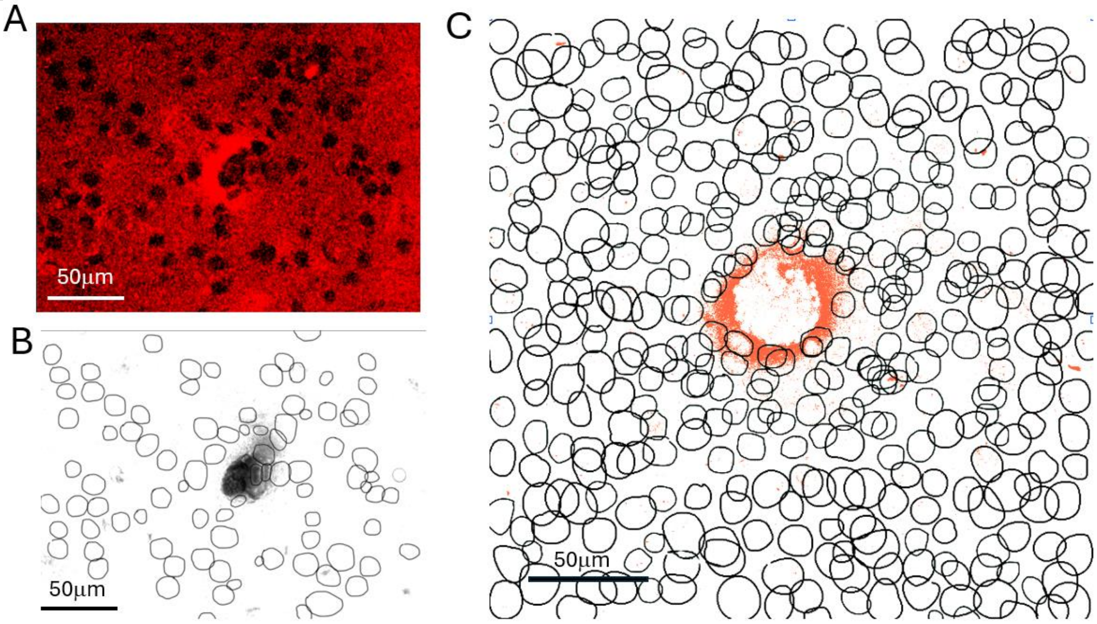

Fig. S1. Histology analysis. A) Example single confocal fluorescence image scan of the tangential 2-2’ slice taken with red (SO) filter. B) ROI for putative neuron somata extracted from A). C) Putative neuron somata extracted from all consecutive confocal fluorescence image scans of the tangential 1-1’ 50μm thick slice.

WILEY-VCH

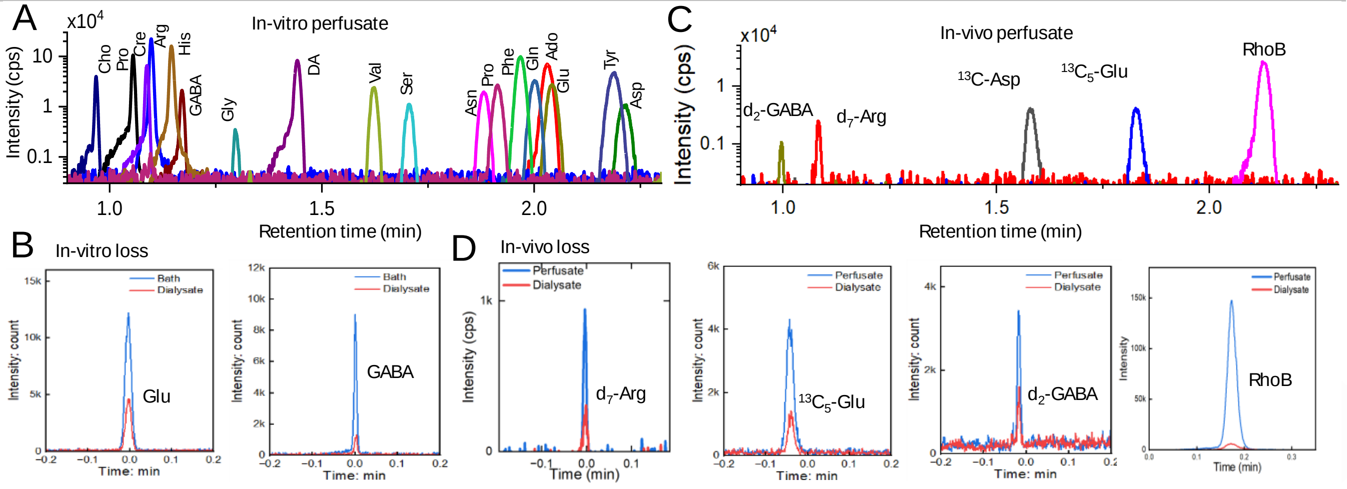

Fig.S2 Characterization of perfusate for in-vitro and in-vivo MS measurements.

A) Electropherograms of reference neurotransmitters and metabolites in a perfusate prepared for in-vitro LOD calibrations and measurements of loss/recovery rates via CE-MS. Semi-log scale. B) CE-MS electropherograms of perfusate for in-vitro recovery measurements for Glu, and GABA, respectively. C) Electropherograms of reference SILS neurochemicals in a perfusate prepared for in-vivo measurements. Semi-log scale. D) CE-MS electropherograms of a perfusate for in-vivo loss measurements for d7-Arg, 13C-Asp, 13C5-Glu, d2-GABA, respectively.

WILEY-VCH
